# The Impact of Phosphorus Supply on Selenium Uptake During Hydroponics Experiment of Winter Wheat (*Triticum aestivum*) in China

**DOI:** 10.3389/fpls.2018.00373

**Published:** 2018-03-20

**Authors:** Hongen Liu, Zhiwei Shi, Jinfeng Li, Peng Zhao, Shiyu Qin, Zhaojun Nie

**Affiliations:** College of Resources and Environment, Henan Agricultural University, Zhengzhou, China

**Keywords:** selenium, chemical forms, phosphorus, subcellular distribution, winter wheat

## Abstract

Selenium (Se) is a necessary trace element for humans and animals, and Se fertilization is an efficient way to increase Se concentration in the edible parts of crops, thus enhance the beneficiary effects of Se in human and animal health. Due to the similarity of physical and chemical properties between phosphate (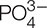) and selenite (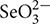), phosphorus (P) supply often significantly impacts the absorption of Se in plants, but little is known about how P supply influences the subcellular distribution and chemical forms of Se. In this study, the effects of P supply on subcellular distribution and chemical forms of Se in winter wheat were investigated in a hydroponic trial with medium Se level (0.1 mg Se L^-1^). P was applied with three concentrations (0.31, 3.1, and 31 mg P L^-1^) in the experiment. The results showed that increasing P supply significantly decreased the concentration and accumulation of Se in the roots, stems, and leaves of winter wheat. An increase in P supply significantly inhibited Se accumulation in the root cell wall, but enhanced Se distribution in the organelles and soluble fraction of root cells. These findings suggest that increased P supply inhibited the root-to-shoot transport of Se. An increase in P supply enhanced Se accumulation in the cell wall of plant stems (both apical and axillary stem) and cell organelles of plants leaves, but inhibited Se distribution in the soluble fraction of stems and leaves. This suggests that P supply enhances Se transportation across the cell membrane in shoots of winter wheat. In addition, increased P supply also altered the chemical forms of Se in tissues of winter wheat. These findings will help in understanding of the regulation grain Se accumulation and provide a practical way to enhance Se intake for humans inform Se-enriched grains.

## Introduction

Selenium (Se) is a necessary trace element for human, and the critical components of glutathione peroxidase (GSH-Px) and prosthetic group of many enzymes (Awasthi et al., 1975; Rotruck et al., 1980). Se plays important roles in antioxidation, detoxification, and high immunity for human beings. Many studies have shown that a low intake of Se may increase the risk of Keshan disease, Kashin–Beck disease, muscle syndrome, liver disease, and many cancers (Liu et al., 2004; Arinola and Charles-Davies, 2008). To increase the intake of Se for human beings, Se supplements in soil or through foliage spray thus producing Se-enriched food from corps have been a popular subject in recent research (He et al., 1998). Although Se is not an essential element for plants, low concentrations of Se have beneficial effects on plant growth and yield. Se was found to elevate antioxidant activity, stimulate starch accumulation in chloroplasts, and ameliorate the toxic effects of several HMs in plants (Pennanen et al., 2002; Turakainen et al., 2004; Malik et al., 2012). However, higher concentrations of Se supply result in toxicity in plants, and there is a narrow boundary between beneficial and toxic concentrations for Se (Kaur et al., 2014). Therefore, a better understanding of the basic mechanism about Se absorption and transport in plants is important to enhance Se accumulation and Se intake in the human diet while not affecting plant growth.

Selenate (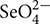) is the main form of Se in alkaline soils, whereas Se exists predominantly as selenite (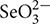) in acid and neutral soils (Elrashidi et al., 1987). Selenite becomes the dominant form in strongly reduced soil (pE + pH < 7.5) (Elrashidi et al., 1987). It has been reported that selenate is similar to sulfate in chemical characteristics, and thus is taken up by plants through sulfate transporters (Anderson, 1993). Furthermore, plants take up Se and phosphorus (P) in the form of anions. Although Se and P are not in the same periodic group, uptake of selenite (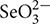) is promoted by P deficiency in wheat, which indicates that selenite (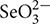) uptake is an active process likely mediated by phosphate transporters (Li H.F. et al., 2008). Earlier studies also pointed out that P could markedly reduce the uptake of Se in plants (Lévesque, 1974; Singh and Singh, 1978). However, conflict results about the effect of P application on the uptake of Se have been reported in plants. Cater et al. (1972) observed that adding P to the soil increased the Se concentrations in alfalfa grown on 6 of 14 soils, and P application increased the Se absorption of berseem when Se supplied from 0 to 16 mg L^-1^ (Singh and Malhotra, 1976). As yet, there have been no reports that describe the chemical pathways in which P affects Se subcellular distribution and chemical form in plants.

Many researchers have studied the subcellular distribution and chemical form of elements in plants (Li et al., 2006; Pan et al., 2016). However, most of these studies were focused on heavy metals, especially Cd and Zn, because the chemical forms and subcellular distribution of heavy metals are closely related to the detoxification mechanism in plants (Li et al., 2006). Vacuole is the most important compartment contributing to the cell soluble fraction (Ramos et al., 2002; Zhang et al., 2013). Deposition in the cell wall and compartmentation to the vacuole are two important mechanisms for detoxification, tolerance, and hyperaccumulation of some heavy metals and trace elements in plants (Zhang et al., 2014). Du et al. (2010) determined Se subcellular distribution in spring tea leaves according to the method used for heavy metals. In view of the important significance of Se biofortification for humans, some studies have determined the Se chemical forms and speciation in plants (Pickering et al., 2003; Ogra et al., 2004; Zhao et al., 2004; Hart et al., 2011), but these studies focused on the Se metabolism compounds (e.g., selenomethionine and selenocystine). Determination of Se chemical forms in plants using the method of sequential extraction is rare, although this method was used to determine the chemical forms of heavy metals in plants. For example, Liu Y. et al. (2010) analyzed the alcohol-soluble, water-soluble, and salt-soluble Se concentrations in spring tea leaves. The effects of Se on growth and physiological metabolism of plants were contradictorily reported because the results were highly dependent on the concentrations and chemical forms of Se used in the assays (Kassis et al., 2007).

Wheat is the main dietary component and the most important source of both calories and protein in many countries (Cakmak, 2008). In China, the Northern Winter Wheat Region contributes about 70% of the national wheat production (Zhuang, 2003). Applying Se-rich fertilizers to improve grain Se content in winter wheat is an effective strategy to increase Se intake for humans in the winter wheat-planting region (Lyons et al., 2003; Li H.F. et al., 2008). It has been reported that excessive Se dietary intake is harmful for humans and animals, which can result in some selenosis symptoms including dermatitis, cracking of nails, hair loss and respiratory distress, and so on (Rayman, 2012). Above 400 μg Se per day for adults can increase the risk of developing Se toxicity (Institute of Medicine, 2000); therefore, control of grain Se concentration in winter wheat is very important for Se nutrition in humans in the north of China. P fertilization is a common agricultural practice to improve grain yield and quality of winter wheat in the north of China. However, excessive P fertilization in intensive agricultural areas results in severe environmental problems such as eutrophication and heavy metal pollution and has recently received great attention in China (Atafar et al., 2010; Le et al., 2010; Li et al., 2014). Policies related to regulation of P application have been highlighted by the Department of Agriculture of China, which helps to meet the key target of balancing crop production and environmental protection (Zhang et al., 2016). Therefore, the influence of P fertilization on Se absorption and translocation in winter wheat is an important issue to be addressed.

The results are expected to enhance our understanding in the interaction of P and Se in plants and provide practical measures for fertilizers containing P and Se that will maintain appropriate concentration of Se in the grain of winter wheat for human health. The objectives of this study were to: (1) validate the effect of P supply on the Se absorption in winter wheat; (2) explore the cellular mechanism of the effect of P on Se uptake and transport in winter wheat; and (3) investigate the transformation of chemical forms of Se in the different tissues of winter wheat in response to different levels of P supply.

## Materials and Methods

### Greenhouse Conditions

According to the method of Nie et al. (2017), a hydroponic experiment was conducted in a greenhouse with controlled environmental condition at about 14/10 h in light/dark regime, 22/18°C in air temperatures, approximate 500 μmol m^-2^ s^-1^ in photon flux density, and 65% in relative humidity.

### Solution Culture

Winter wheat (*Triticum aestivum* cv. Wenmai 8) seeds, obtained from Henan Qiule Seeds Company (Zhengzhou, China), were germinated in deionized water at 25°C for 5 d, after sterilized in 0.5% NaClO solution for 15 min. Then, 20 seedlings were transferred to 4 L plastic containers with Hoagland–Arnon nutrition solutions (Arnon and Hoagland, 1940), which was consisted of 945 mg L^-1^ Ca(NO_3_)_2_⋅4H_2_O, 607 mg L^-1^ KNO_3_, 493 mg L^-1^ MgSO_4_⋅7H_2_O, 20 mg L^-1^ EDTA-Fe, 2.86 mg L^-1^ H_3_BO_3_, 1.81 mg L^-1^ MnCl_2_⋅4H_2_O, 0.22 mg L^-1^ ZnSO_4_⋅7H_2_O, 0.08 mg L^-1^ CuSO_4_⋅5H_2_O, and 0.02 mg L^-1^ (NH_4_)_6_Mo_7_O_24_⋅4H_2_O. The final pH of the solution was adjusted to 6.0. P was added to the solutions as NaH_2_PO_4_ at three rates: 0.31, 3.1, and 31 mg P L^-1^ and Se was added as Na_2_SeO_3_ at 0.1 mg Se L^-1^ (Liu et al., 2004; Liu H. et al., 2010). Three times was replicated in each treatment. Seedlings were supplied full-strength nutrient solutions until sampled, except for the quarter and half strength solutions supplied at the first and second weeks, respectively. The solutions were replaced every 3 days to ensure enough nutrients. Solution of 5% HCl was used to dip all vessels for 1 week, and then deionized water was used to wash those vessels more than three times. After cultivation for 21 d, the roots, stems, and leaves of 14 seedlings were separately sampled, dried at 70 ± 5°C, and analyzed for dry weights and concentrations of P and Se. The leaves, stems, and roots of other six seedlings were immediately frozen in liquid nitrogen, and then stored at -20°C for further subcellular fractions and chemical forms analysis. All the procedures about preparation and renewal of nutrient solution and the harvest of plants samples were conducted according to the method of Nie et al. (2017).

### Subcellular Fractions Analysis

The subcellular fractions of Se were analyzed according to the method of Weigel and Jäger (1980). About 1 g of frozen samples (leaf, stem, or root) was placed into 50 mL polypropylene centrifuge tubes and was homogenized in 20 mL extraction buffer containing 50 mM Tris–HCl (pH 7.5), 250 mM sucrose, and 1.0 mM dithioerythritol. The cell wall fraction was obtained in the residue after centrifuging the homogenate at 300 × *g* for 30 s. The cell organelle fraction was in retained pellet after centrifuging the supernatant at 10,000 × *g* for 30 min. The resultant supernatant solution was regarded as soluble fraction. All steps were performed at 4°C.

### Chemical Forms Analysis

The chemical forms of Se in the different plant issues were determined according to the method of Liu Y. et al. (2010). The extractive solutions were in the following order: (1) 80% ethanol, extracting monosaccharides, two sugars, and small molecules organic Se dissolved in ethanol soluble. (2) Deionized water (d-H_2_O), extracting water soluble protein and sugar-integrated Se, and inorganic Se. (3) 1 M NaCl, extracting salt soluble protein-integrated Se and exchangeable Se substance with salt. (4) 2% Acetic acid (HAc), extracting Se–phosphate complexes. (5) 0.6 M HCl, extracting Se oxalate. (6) Residual Se. A 0.5 g portion of frozen leaf, stem, or root samples were homogenized in the above extractive solutions with a diluted ratio at 1:100 (w/v), and then shaken at 25°C for 22 h. After that, the first supernatant solution was obtained after centrifuging the homogenate at 5000 × *g* for 10 min, and placed into a conical flask. The sedimentation was re-suspended by the same extractive solution for twice and repeated the centrifuge steps for each extraction solutions. The supernatant of the three suspending was then pooled.

### Mineral Analysis

Selenium concentration was analyzed according to the method of Chinese Standard (GB/T 21729, 2008). The ground dry samples were digested with 5 mL of HNO_3_: HClO_4_ (4:1, v/v) until white smoke appeared. And then, 10 mL 6 mol L^-1^ HCl was added into the digestive system until the white smoke appeared again. The residual was then diluted with 25 mL deionized water. The cell wall, cell organelle, and soluble fractions of tissues, the extraction solutions of five chemical forms, and residual were transferred to conical flasks to dry by distillation, and digested as described above. The concentrations of Se in digested solutions were determined using Atomic Fluorescence Spectrometer (AFS8220, Beijing Jitian Instruments Company, China). The details for Se subcellular fractions and chemical forms analysis were: limits of detection 0.01 μg L^-1^, recovery rate 85.4–98.5%.

The P concentration was determined according to the method of Bao (2002). Ground samples were digested with 10 mL of H_2_SO_4_ and several drops of H_2_O_2_, and then diluted with deionized water. The sample solution was determined using the vanadomolybdo-phosphoric yellow colorimetric method using a FOSS FIAstar 5000 Autoanalyzer.

### Calculations and Statistical Analysis

The Se accumulation was calculated with the Se concentration, multiplied by the dry weights.

The proportion (%) of subcellular fractions (or chemical form) of Se was calculated as the percentage of Se concentrations in each fraction (forms) to all fractions (forms), according to the method of Nie et al. (2017).

According to the method of Erenoglu et al. (2011), all data were statistically analyzed by one-way ANOVA with LSD multiple comparison at a 5% level (*P* < 0.05) using SPSS 18.0 software.

## Results

### Dry Weights, Se Concentrations, and Accumulation

According to the results of one-way ANOVA, P application exerted significant effects (*P* < 0.05) on dry weights, Se concentrations, and accumulation in tissues of winter wheat (Supplementary Table S1). At three concentrations of P supply, P_0.31_, P_3.1_, and P_31_, the dry weight of roots significantly increased comparing P_3.1_ with P_0.31_, while the root dry weight decreased comparing P_31_ to P_0.31_ (*P* < 0.05). The dry weights of stems and leaves remarkably increased with P_3.1_ comparing to P_0.31_ (*P* < 0.05), but no significant differences between the treatments of P_3.1_ and P_31_ (**Table 1**).

**Table 1:** Dry weights, Se concentration, and accumulation in tissues of winter wheat (*Triticum aestivum* cv. Wenmai 8) seedlings, pre-cultured with 0.31, 3.1, and 31 mg P L^-1^ in a nutrient solution for 21 d.

Tissues	Treatment	Dry weights (g pot^-1^)	Se concentration (mg kg^-1^)	Se accumulation (μg pot^-1^)
Roots	P_0.31_	0.22 ± 0.01ab	469 ± 8.66a	102 ± 2.58a
	P_3.1_	0.27 ± 0.03a	171 ± 18.4b	45.6 ± 6.97b
	P_31_	0.17 ± 0.02b	33.1 ± 0.86c	5.49 ± 0.61c
Stems	P_0.31_	0.04 ± 0.00b	36.8 ± 1.05a	1.47 ± 0.04b
	P_3.1_	0.16 ± 0.03a	27.0 ± 1.08b	4.23 ± 0.81a
	P_31_	0.13 ± 0.01a	3.39 ± 0.24c	0.45 ± 0.06b
Leaves	P_0.31_	0.17 ± 0.01b	54.9 ± 7.31a	26.0 ± 5.74a
	P_3.1_	0.46 ± 0.05a	24.6 ± 2.07b	4.17 ± 0.36b
	P_31_	0.43 ± 0.04a	7.30 ± 0.66c	3.20 ± 0.55b

*Values are means of three independent replicates. For each trait, means followed by different letters are significantly different from each other according to one-way ANOVA followed by least significant difference (LSD) multiple comparison (*P* < 0.05)*.

Selenium concentrations in roots, stems, and leaves were sharply decreased (*P* < 0.05) with increased P supply. Increasing P supply also significantly decreased (*P* < 0.05) Se accumulation in roots and leaves. Se accumulation in stems in the treatment of P_3.1_ was higher than that in the treatment of P_0.31_ and P_31_.

### Se Subcellular Fraction and Distribution

One-way ANOVA results revealed significant effect of P application on subcellular fractions of Se in tissues of winter wheat (Supplementary Table S2; *P* < 0.05). According to **Table 2**, most of Se accumulated in the cell wall and cell organelle in stems and leaves, with less Se present in the soluble fraction. However, Se accumulation in each fraction in roots depended on the P supply levels. In roots, most of Se accumulated in the cell wall in the treatment of P_0.31_, while Se accumulated in the soluble fraction in the treatment of P_31_.

**Table 2:** Subcellular fractions of Se in tissues of winter wheat (*T. aestivum* cv. Wenmai 8) seedlings, pre-cultured with 0.31, 3.1, and 31 mg P L^-1^ in a nutrient solution for 21 d (Unit: mg kg^-1^ FW).

Tissues	Treatment	Cell wall	Cell organelle	Soluble fraction
Roots	P_0.31_	79.6 ± 5.84a	9.48 ± 1.86a	11.8 ± 2.28a
	P_3.1_	0.97 ± 0.07b	0.77 ± 0.09b	0.39 ± 0.11b
	P_31_	0.04 ± 0.00b	0.10 ± 0.01b	0.47 ± 0.02b
Stems	P_0.31_	2.30 ± 0.03a	1.11 ± 0.21a	0.25 ± 0.04a
	P_3.1_	2.04 ± 0.47a	0.52 ± 0.04b	0.17 ± 0.02a
	P_31_	0.20 ± 0.01b	0.03 ± 0.01c	0.00 ± 0.00b
Leaves	P_0.31_	3.36 ± 0.38a	0.64 ± 0.15a	0.18 ± 0.05a
	P_3.1_	2.14 ± 0.15b	0.45 ± 0.02a	0.02 ± 0.00b
	P_31_	0.24 ± 0.01c	0.06 ± 0.01b	0.00 ± 0.00b

*Values are means of three independent replicates. For each trait, means followed by different letters are significantly different from each other according to one-way ANOVA followed by LSD multiple comparison (*P* < 0.05)*.

Selenium concentration in each fraction of three tissues all showed a strong decrease (*P* < 0.05) with increasing P supply levels, with the lowest Se concentration in the treatment of P_31_ (**Table 2**). However, there were no obvious differences of Se concentration in each fraction of roots, and in the soluble fractions of leaves between the treatment of P_3.1_ and P_31_. No pronounced differences were observed in Se concentrations in the cell wall and soluble fractions of stems, and in cell organelles of leaves between the treatment of P_0.31_ and P_3.1_.

**FIGURE 1:**
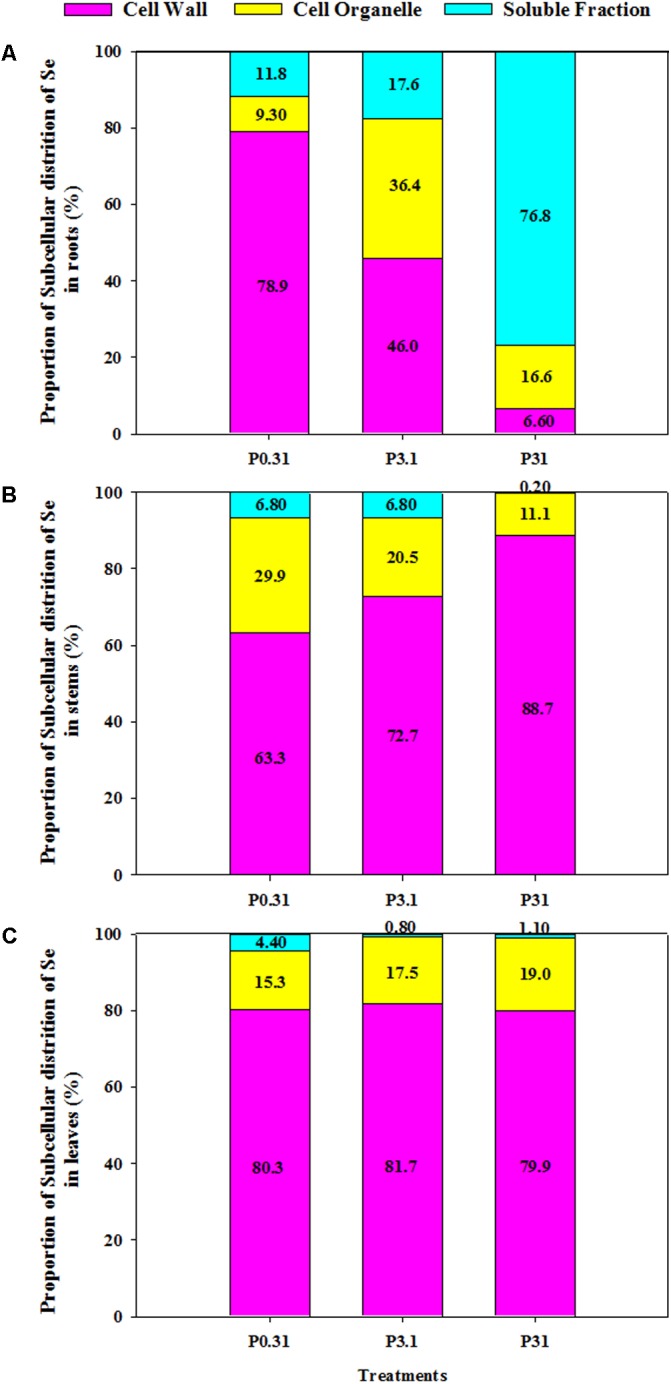
Proportion of subcellular distribution of Se in roots **(A)**, stems **(B)**, and leaves **(C)** of winter wheat (*Triticum aestivum* cv. Wenmai 8) seedlings, grown at 0.31, 3.1, or 31 mg P L^-1^ in a nutrient solution for 21 d. The proportion of subcellular distribution of Se (%) was calculated as the percentage of Se concentrations in each fraction to all fractions in each tissue. Values are means of three independent replicates.

The proportion of Se in the cell wall was higher than the cell organelles or soluble fraction, except for the major portion of soluble fractions in roots at P_31_ treatment (**Figure 1**). Se proportion in the cell wall of roots was strongly decreased by increasing P supply; however, an increase in Se in the cell organelle and soluble fraction was observed in roots (**Figure 1A**). On the contrary, increasing P supply levels significantly increased Se proportion in the cell wall fractions of stems, but decreased Se proportion in cell organelle and soluble fractions of stems (**Figure 1B**). The Se proportion in the cell organelle of leaves in the treatment of P_0.31_ was lower than that in the treatment of P_3.1_ or P_31_; however, Se proportion in the soluble fraction of leaves in the treatment of P_0.31_ was higher than that in the treatment of P_3.1_ or P_31_ (**Figure 1C**). The P supply levels had no effect on Se proportion in the cell wall fraction of leaves.

### Se Chemical Forms and Distribution

**FIGURE 2:**
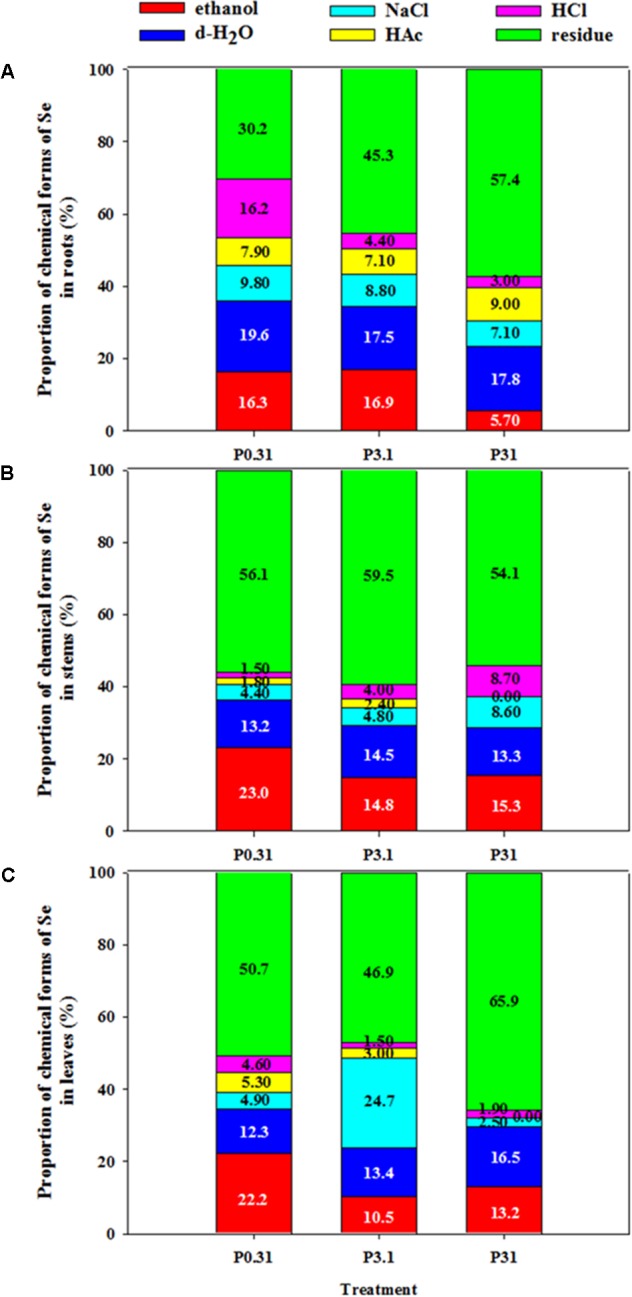
Proportion of chemical forms of Se in roots **(A)**, stems **(B)**, and leaves **(C)** of winter wheat (*T. aestivum* cv Wenmai 8) seedlings, grown at 0.31, 3.1, or 31 mg P L^-1^ in a nutrient solution for 21 d. The proportion of chemical forms of Se (%) was calculated as the percentage of Se concentrations in each form to all forms in each tissue. Values are means of three independent replicates.

According to the results of one-way ANOVA, P application had significant effects (*P* < 0.05) on chemical forms of Se in tissues of winter wheat (Supplementary Table S3). Residual, 80% ethanol, and deionized water extractive forms of Se were dominant in each treatment in winter wheat (**Table 3** and **Figure 2**).

**Table 3:** Chemical forms of Se in tissues of winter wheat (*T. aestivum* cv. Wenmai 8) seedlings, pre-cultured with 0.31, 3.1, and 31 mg P L^-1^ in a nutrient solution for 21 d (Unit: mg kg^-1^ FW).

Tissues	Treatment	Ethanol	d-H_2_O	NaCl	HAc	HCl	Residue
Roots	P_0.31_	3.67 ± 0.10a	4.42 ± 0.50a	2.21 ± 0.25a	1.78 ± 0.06a	3.64 ± 0.18a	6.79 ± 0.23a
	P_3.1_	0.69 ± 0.05b	0.71 ± 0.05b	0.36 ± 0.03b	0.28 ± 0.03b	0.18 ± 0.04b	1.84 ± 0.12b
	P_31_	0.05 ± 0.01c	0.13 ± 0.05b	0.05 ± 0.02b	0.07 ± 0.01c	0.02 ± 0.01b	0.47 ± 0.12c
Stems	P_0.31_	1.07 ± 0.11a	0.62 ± 0.12a	0.21 ± 0.04a	0.08 ± 0.02a	0.07 ± 0.01a	2.60 ± 0.21a
	P_3.1_	0.33 ± 0.01b	0.33 ± 0.07ab	0.11 ± 0.02ab	0.05 ± 0.01ab	0.09 ± 0.04a	1.34 ± 0.11b
	P_31_	0.09 ± 0.01c	0.08 ± 0.05b	0.05 ± 0.02b	0.00 ± 0.00b	0.05 ± 0.02a	0.32 ± 0.07c
Leaves	P_0.31_	1.05 ± 0.05a	0.61 ± 0.32a	0.24 ± 0.13a	0.24 ± 0.10a	0.24 ± 0.21a	2.39 ± 0.27a
	P_3.1_	0.35 ± 0.00b	0.52 ± 0.19a	0.18 ± 0.03a	0.09 ± 0.05ab	0.07 ± 0.03a	1.58 ± 0.25a
	P_31_	0.11 ± 0.00c	0.13 ± 0.13b	0.02 ± 0.02b	0.00 ± 0.00b	0.02 ± 0.01a	0.59 ± 0.24b

*Values are means of three independent replicates. For each trait, means followed by different letters are significantly different from each other according to one-way ANOVA followed by LSD multiple comparison (*P* < 0.05)*.

Selenium forms extracted by 80% ethanol in each tissue, Se forms extracted by deionized water and 2% HAc in roots, and residual Se in roots and stems decrease (*P* < 0.05) significantly but gradually with increasing P supply levels, with the lowest Se in the treatment of P_31_ (**Table 3**). Meanwhile, P_0.31_ had higher Se forms extracted by 1 M NaCl in roots as well as 6 M HCl in roots and leaves than the treatment of P_3.1_ and P_31_. Se forms extracted by deionized water, 1 M NaCl, and 2% HAc in stems and leaves in the treatment of P_0.31_ were also higher than that in the treatment of P_31_. Compared to the treatment of P_0.31_, residual Se in leaves of the treatment of P_31_ significantly decreased (*P* < 0.05).

The higher proportion of residual Se than other forms was found in each tissue of winter wheat (**Figure 2**). In roots, increasing P supply levels significantly increased Se proportion in residue, but decreased Se proportion in 80% ethanol and 6 M HCl (**Figure 2A**). In stems, Se proportion in 80% ethanol showed a strong decrease, but that in 1 M NaCl and 6 M HCl showed a strong increase with an increase in P supply levels (**Figure 2B**). In leaves, increasing P supply also significantly decreased Se proportion in 80% ethanol, 2% HAc, and 6 M HCl, but increased Se proportion in deionized water and residue (**Figure 2C**). In addition, P_3.1_ had the higher Se proportion in residue of stems and in 1 M NaCl of leaves, but lower Se proportion in residue of leaves than the treatment of P_0.31_ and P_31_.

## Discussion

Earlier reports showed that Se concentration and accumulation were dependent on the status of P nutrition in plants (Cater et al., 1972; Lévesque, 1974; Singh and Malhotra, 1976; Singh and Singh, 1978; Altansuvd et al., 2014; Zhang et al., 2017). As expected, the current study results clearly indicated that increasing P supply levels greatly decreased Se concentration and accumulation in roots, stems, and leaves of winter wheat (**Table 1**).

There are contradictory opinions on the effect of P application on the concentration and accumulation of Se in plants. Zhang et al. (2017) suggested that P combined with selenite application declined the Se accumulation coefficient, Se translocation coefficient, and Se concentration in tissues of wheat, but when P was applied with selenate (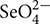), the Se content, Se accumulation coefficient, and Se translocation coefficient in wheat all increased. This indicates that the effect of P application on Se concentration and accumulation depended on the type of Se fertilizer. The current study, using selenite (SeO_3_^2^) as the Se fertilizer, is consistent with the results of Zhang et al. (2017). In contrast, Altansuvd et al. (2014) pointed out that the Se content of potato, wheat, and barley grown in high available P soil was higher than those in low available P soil, and total soil Se was significantly correlated with P availability in soil. During previous studies (Mora et al., 2008; Sanghun et al., 2011), it was observed that moderate P supply combined with Se fertilization could increase grain yield while P application had a negative effect on increasing grain Se concentration, thus avoiding excessive P application in agricultural practice is critical for the balance of obtaining higher grain yield and appropriate grain Se concentration in plants. It was also observed that the interaction of P and Se in plants often focused on Se absorption and translocation, but less attention has been paid to the Se subcellular distribution and chemical form transformation (Mora et al., 2008; Sanghun et al., 2011).

The subcellular distribution pattern of elements correlates to their changes during growth and their biochemical or structural roles in plants (Rathore et al., 1972). Under toxic concentrations, plants roots inhibit the translocation of these elements to shoots as a barrier (Weigel and Jäger, 1980). A general hypothesis is that the cell walls and vacuoles in root are the major sites for sequestrating elements, and distribution of elements in subcellular fractions plays an important role in plant tolerance to the toxic levels of elements (Hall, 2002; Gallego et al., 2012). In the current study, the majority of Se in roots was accumulated in the cell wall fraction (78.9 and 46.0%) at low (P_0.31_) and medium P (P_3.1_) application, whereas a minor fraction was accumulated in the cell wall (6.58%) when P supplied at 31 mg L^-1^. This suggests that the subcellular distribution pattern of Se in roots may be dependent on P concentrations in the medium.

As the first cellular barrier, the cell wall prevents the entrance of ions into cells through ions binding and retention, due to its negative charge (Gallego et al., 2012). The current study results also showed that an increase in P supply levels significantly decreased Se concentration and distribution in the cell wall of roots (**Table 2** and **Figure 1A**). It indicated that P supply resulted in cell wall bind less Se ions and enhanced Se transportation across the cell membrane (Weigel and Jäger, 1980). Enhanced accumulation of heavy metal ions (such as Cd^2+^) in shoot was contributed by heavy metal ions distributing in the cell wall (Su et al., 2014), thus inhibited Se distribution in cell wall by an increase in P supply might be one of the reasons for decreased Se accumulation in tissues of winter wheat by an increase in P supply (**Table 1**). On the other hand, most of heavy metal ions enter into the vacuoles of root cells to be compartmentalized, which are considered as a detoxification mechanism of heavy metals due to the reduced heavy metal ions concentration in the cytoplasm (Su et al., 2014). The present study found that an increase in P supply decreased Se concentration in the cell organelle and soluble fraction of roots, but enhanced Se distribution in the cell organelle and soluble fractions (**Figure 1A**). The large proportion of Se distributing in the vacuoles means that Se entrance into the xylem from the root cells, and thus translocation to shoots was limited (Nocito et al., 2011). In contrast, an increase in P supply enhanced Se distribution in the cell wall of stems, but inhibited Se distribution in the cell organelle and soluble fraction of stems (**Figure 1B**). The current results also suggested that P supply enhanced Se transportation across the cell membrane and transported to the leaves. In leaves, an increase in P supply decreased Se distribution in the soluble fraction, but increased in the cell organelle fraction (**Figure 1C**). These results suggest that the increasing P supply may enhance metabolism related to Se by increasing distribution of Se in the cell organelle (Winkel et al., 2015).

Plant Se metabolism and transformation are important for Se nutrition of humans, because human dietary Se are mainly from plants. In addition, the beneficial or toxic effect of Se on plant growth and physiological metabolism is closely related to the chemical forms of Se in plants (Beilstein et al., 1991). Root-to-shoot or stem-to-leaf translocation of Se and the bioavailability of Se in plants are also dependent on its chemical forms in plant tissues (Beilstein et al., 1991; Kassis et al., 2007; Su et al., 2014). The present study found that most of Se in roots, stems, and leaves of winter wheat is in the form of residual Se (30.2–65.9%), followed by the monosaccharides, two sugars, and small molecules organic Se (5.70–23.0%, extracted by 80% ethanol), water-soluble protein and sugar integrated Se, as well as inorganic Se (12.3–19.6%, extracted by deionized-H_2_O), soluble protein integrated Se, and exchangeable Se substance with salt (4.44–24.75%, extracted by 1 M NaCl) (**Table 3** and **Figure 2**). Liu Y. et al. (2010) and Li F. et al. (2008) also reported that Se chemical forms extracted by 80% ethanol, deionized water, and 1 M NaCl predominated in plant issues of Se-enriched tea. Increasing P supply obviously decreased Se concentration in each chemical forms in roots, stems, and leaves of winter wheat.

An increase in P supply improved Se proportion in the form of residue, but resulted in a decrease in the Se form extracted by 80% ethanol in all tissues (**Table 3** and **Figure 2**), which suggests that the increase of P supply can inhibit Se bioavailability in winter wheat. With P supply levels increased, Se form extracted by 6 M HCl in roots and leaves decreased. The 6 M HCl-extractable Se consisted of Se-oxalate complexes in plant issues, which are the inert metabolism substances in plants (Libert and Franceschi, 1987). Under the condition of lower P supply levels, increasing the proportion of the 6 M HCl-extractable Se may result in decrease in Se phyto-availability, and have a role in alleviating the Se toxicity in plants issues. In addition, with an increase in P supply levels, the Se form extracted by 1 M NaCl in roots decreased, but that in stems and leaves increased. The Se form extracted by 1 M NaCl contains salt soluble protein-integrated Se and exchangeable Se substance with salt (Liu Y. et al., 2010). The Se is assimilated into Se–amino acids complexes to perform its normal physiological function after absorbing by plants (Kaur et al., 2014). These results suggest that the Se forms extracted by 1 M NaCl in stems and leaves due to an increase in P supply levels may play an positive role in Se physiological metabolism in shoots of winter wheat. Moreover, the increasing P supply had different influences on the other forms of Se in various tissues. These differences may be due to the different role of P and Se in diverse plant tissues.

Selenium biofortifications are often through the edible parts of crops to supplement Se nutrition for humans. In the previous study (Sanghun et al., 2011), it was found that moderate application of P fertilizer combined with Se application had a positive effect on grain yield, but excessive P fertilization had a negative effect on Se absorption and grain Se concentration in winter wheat. The current study results imply that avoiding excessive P fertilization is conducive to maintain the balance of obtaining greater grain yield and grain Se concentration in winter wheat in agricultural practices. The current study only investigated the Se uptake and chemical transformation in the vegetative parts of winter wheat in response to the levels of P supply.

## Conclusion

The current study would provide better understandings on the mechanism of P application on Se absorption and transformation in winter wheat, and could assist in agricultural practices for increasing grain Se concentration and grain yield of winter wheat to supplement Se nutrition safely for humans and animals. The current study demonstrated that an increase in P supply inhibited Se accumulation in winter wheat that increasing P supply significantly decreased Se concentration and accumulation in roots, stems, and leaves in winter wheat. An increase in P supply significantly inhibited Se distribution in root cell wall and shoots (including stems and leaves) soluble fraction, but enhanced Se distribution in root cell organelle, root soluble fraction, stem cell wall, and leaf cell organelle. Therefore, the current study results suggest that increasing P supply has negative effect on the transport of Se from root to shoot, and enhanced Se transportation across the cell membrane. Increased P supply alters chemical forms of Se in tissues of winter wheat, indicating that P supply influences the bioavailability of Se differently in various tissues.

## Author Contributions

ZN conceived and designed the experiments. ZS and JL performed the experiments. PZ and SQ analyzed the data. HL wrote the paper.

## Conflict of Interest Statement

The authors declare that the research was conducted in the absence of any commercial or financial relationships that could be construed as a potential conflict of interest.
