# Supplementary material for: The Impact of Phosphorus Supply on Selenium Uptake During Hydroponics Experiment of Winter Wheat (Triticum aestivum) in China
Source: Front Plant Sci. 2018 Mar 20;9:373. doi: 10.3389/fpls.2018.00373 (PMC5869179; doi:10.3389/fpls.2018.00373)
Supplement: Supplementary file 1 [file Table_1.DOC]

**Supporting Information Tables S1–S3**

**Table S1** One-way analysis of variance (ANOVA) of the effect of P application on the dry weights, Se concentration and accumulation in tissues of winter wheat (*Triticum aestivum* cv Wenmai 8) seedlings, pre-cultured with 0.31, 3.1 and 31 mg P L^-1^ in a nutrient solution for 21 d.

| Source of Variation (Treatment) | Tissue | F_Dry weights_ | F_Se concentration_ | F_Se accumulation_ |
| --- | --- | --- | --- | --- |
| P treatment | roots | 5.49* | 359** | 125** |
|  | stems | 11.6** | 378** | 17.3** |
|  | leaves | 18.7** | 29.9** | 14.9** |

**Table S2** One-way analysis of variance (ANOVA) of the effect of P application on the subcellular fractions of Se in tissues of winter wheat (*Triticum aestivum* cv Wenmai 8) seedlings, pre-cultured with 0.31, 3.1 and 31 mg P L^-1^ in a nutrient solution for 21 d.

| Source of Variation (Treatment) | Tissue | F_Cell wall_ | F_Cell organelle_ | F_Soluble fraction_ |
| --- | --- | --- | --- | --- |
| P treatment | roots | 184** | 23.8** | 24.8** |
|  | stems | 17.7** | 19.1** | 20.9** |
|  | leaves | 43.8** | 11.7** | 12.9** |

**Table S3** One-way analysis of variance (ANOVA) of the effect of P application on the chemical forms of Se in tissues of winter wheat (*Triticum aestivum* cv Wenmai 8) seedlings, pre-cultured with 0.31, 3.1 and 31 mg P L^-1^ in a nutrient solution for 21 d.

| Source of Variation (Treatment) | Tissue | F_Ethanol_ | F_d-H2O_ | F_NaCl_ |
| --- | --- | --- | --- | --- |
| P treatment | roots | 951** | 64.4** | 64.8** |
|  | stems | 60.7** | 9.85* | 8.19* |
|  | leaves | 256** | 31.5** | 35.7** |
| Source of Variation (Treatment) | Tissue | F_HAc_ | F_HCl_ | F_Residue_ |
| P treatment | roots | 582** | 1028** | 408** |
|  | stems | 7.07* | 0.61 | 62.7** |
|  | leaves | 3.68 | 0.92 | 12.7** |
